# Geographic Variation in Host-Specificity and Parasitoid Pressure of an Herbivore (Geometridae) Associated with the Tropical Genus *Piper* (Piperaceae)

**DOI:** 10.1673/031.009.2801

**Published:** 2009-06-02

**Authors:** Heidi Connahs, Genoveva Rodríguez-Castañeda, Toni Walters, Thomas Walla, Lee Dyer

**Affiliations:** ^1^ 400 Boggs Ecology and Evolutionary biology, Tulane University, New Orleans, LA 70118; ^2^ Mesa state College, 1100 North Avenue, Grand Junction, Colorado 80501; ^3^ Biology Department, University of Nevada; Reno, Nevada

**Keywords:** Eois, caterpillars, diet breadth, parasitism, tropical forests, precipitation, elevation, Costa Rica, Panama, Ecuador

## Abstract

The extraordinary diversity of tropical herbivores may be linked to hostplant specialization driven in part by variation in pressure from natural enemies. We quantified levels of host-specificity and parasitoid attack for the specialist herbivore, *Eois* (Geometridae). The goals of this research were to examine: 1) whether *Eois* are specialized on the genus *Piper* (Piperaceae) and if hostplant specialization varies geographically; 2) whether *Eois* are equally vulnerable to parasitoid attack across different geographic regions and by the same parasitoid families; and 3) whether parasitism levels vary with precipitation and elevation. Based on over 15,000 rearings, we found *Eois* caterpillars feeding exclusively on *Piper*. However, we did not detect geographic differences in host-specificity; each *Eois* species fed on an average of two *Piper* species. Parasitism levels of *Eois* varied significantly with climate and topography; *Eois* were most vulnerable to parasitoid attack in moist versus dry and wet forests and at low versus high elevations. The diversity of parasitoid families reared from *Eois* was greater in Ecuador and Costa Rica than in Panama, where parasitoids were primarily in the family Braconidae. The quantitative evidence for host-specificity provides support for the hypothesis that *Eois* are specialized on *Piper*. Our results also reveal that *Eois* are exposed to a mosaic of potential selective pressures due to variation in parasitoid attack over a large spatial scale.

## Introduction

A major goal of evolutionary ecology is to identify selective forces driving the evolution of specialization (Ehrlich and Raven 1964; Bernays and Graham 1988; Jaenike 1990). Most plant-feeding insects are relatively specialized, feeding on a few related plant genera or on plants belonging in a single family (Bernays and Graham 1988; Gratton and Welter 1999). Numerous hypotheses have been proposed to explain the advantages of specialization. In their seminal paper, Ehrlich and Raven (1964) postulated that herbivore specialization is an adaptation to specific host-plant secondary metabolites. An alternative view is that specialist herbivores benefit by escaping natural enemies or sequestering toxic plant compounds for their own defenses (Bernays and Graham 1988; Dyer 1995; Gratton and Welter 1999; Termonia et al. 2002; Vend et al. 2005). Causal explanations of high herbivore specificity in the tropics are not well developed, but it is widely accepted that top-down and bottom-up forces can act in concert to influence herbivore populations and diet breadth (Price et al. 1980; Hunter and Price 1992; Forkner and Hunter 2000; Denno et al. 2005). One hypothesis to explain the putative high proportion of specialized insect herbivores focuses on tritrophic interactions and the concept that variation in plant defenses and pressure from natural enemies drive herbivores to specialize (Dyer 1995; Gratton and Welter 1999; Dyer and Coley 2002; Singer and Stireman 2005; Dyer et al. 2007). Empirical evidence suggests that pressure from natural enemies is more intense in the tropics (Jeanne 1979; Dyer and Coley 2002) and tropical plants are chemically ‘nastier’ due to a higher diversity of secondary compounds (Miller and Hanson 1989; Gauld and Gaston 1994; Coley and Barone 1996; Dyer and Coley 2002). Thompson (1988) proposed that geographic variation in trophic interactions could lead to locally adapted populations that follow unique and divergent evolutionary trajectories. Here, we argue that geographic variation in hostplant quality and natural enemy attack may provide a patchwork of selective pressures that promote herbivore specialization in tropical habitats.

If latitudinal gradients in diversity and specialization exist, then tropical forests are likely to be cradles of highly specialized interactions (McKenna and Farrell 2006). Within the tropics, geographic regions can include a variety of climatic conditions and are often composed of a mosaic of plant and animal assemblages, ranging from lowland dry and wet forests to montane tropical cloud forests. In the Neotropics, such topographic diversity and climatic variation can produce heterogeneous selective pressures across relatively short geographic distances. For example, plants in high rainfall forests are thought to be better defended against herbivores than plants in drier forests, due to greater leaf longevity, mechanical toughness and lower nutritional value (Coley and Aide 1991). It is also possible that natural enemies increase with greater rainfall and reduced climate variability, causing lower herbivore density in wetter tropical forests (Coley and Barone 1996; Stireman et al. 2005; Dyer 2007). Changes in plant characteristics along rainfall or altitudinal gradients might have important effects on herbivore specialization and potential coevolutionary relationships with their hostplants and natural enemies.

It has been argued that the traditional approach for investigating the importance of top-down and bottom-up forces on herbivore populations has been unidimensional, with most studies focusing on a single site (Gripenberg and Roslin 2007). The goal of this study was to take a broad spatial approach to examine variation in measures of host specificity for larvae of the common and widely distributed genus *Eois* (Geometridae), an herbivore that utilizes the species rich plant genus *Piper* (Piperaceae). Further, we examined the incidence of parasitoid attack for *Eois* with changes in elevation and precipitation, as well as across geographic regions. Evidence for variation in parasitism and host specificity lends support to the hypothesis that specialization in the tropics is driven by spatial variation in tritrophic interactions.

To investigate spatial variation in host specificity and parasitism of *Eois*, we used data sets including more than 15,000 individual *Eois* caterpillars, reared either to adult or parasitoid, from forests in Costa Rica, Panama and Ecuador. Furthermore, we quantified hostplant specificity using two measures and compared levels of *Piper* specialization across geographic regions. Specifically, we addressed the following questions: 1) How specialized are *Eois* and does this specialization vary geographically? 2) Is there geographic variation in parasitism rates of *Eois?* 3) Do parasitism rates vary with elevation and precipitation; and 4) Are *Eois* vulnerable to attack from the same parasitoid families at different sites? Based on the known natural history of this system, as well as on theoretical considerations, we made the following predictions: 1) *Eois* are highly specialized on particular species within the genus *Piper*, 2) Herbivore specialization and parasitism rates vary geographically; 3) Parasitism is higher in wet forests due to greater plant defenses that prolong caterpillar development, thus increasing their exposure to parasitoids (Benrey and Denno 1997); 4) Parasitism will decrease with elevation due to the negative effects of cold temperatures on parasitoids (Mueller and Schmid-Hempel 1993); and 5) *Eois* will be attacked by the same parasitoid families at each site.

## Materials and Methods

### Study system

Piperaceae is one of the most diverse plant families in the Neotropics and includes *Piper*, which ranks among the top five species-rich genera in tropical rain forests (Gentry 1993; Greig 2004; Jaramillo 2006). *Piper* has a pantropical distribution with nearly 2000 described species though its greatest diversity is found in Neotropical lowland forests (Jaramillo 2006, Quijano-Abril et al. 2006) particularly in the wet areas of the Andes (Greig 2004; Marquis 2004). Relatively few species of *Piper* are widely distributed and it has been suggested that their fragmented distribution patterns are related to historical tectonic events (Quijano-Abril et al. 2006). Plants in this genus exhibit a diverse range of growth forms, including vines, herbs, shrubs and small trees and occur in a wide range of habitats, from highly disturbed areas to the deep shade of the understory, most often in moist habitats Jaramillo 2006; Tebbs 1989). *Piper* species contain a high diversity of phytochemicals; in the 112 species that have been investigated, researchers have identified 667 different secondary metabolites, many of which are deterrent to herbivores (Dyer et al. 2004a). Some of these compounds, such as imides and amides, are toxic to generalist herbivores (Dyer et al. 2003, 2004b); certain *Piper* species can produce up to 37 different imides/amides (Dyer et al. 2004a). The remarkable diversity of *Piper* compounds may thus exert strong pressure on herbivores to specialize (Dyer et al. 2003). *Piper* has been the focus of considerable research (reviewed by Dyer and Palmer 2004), and the arthropod communities associated with this genus show great potential as model study systems.

**Table 1.**
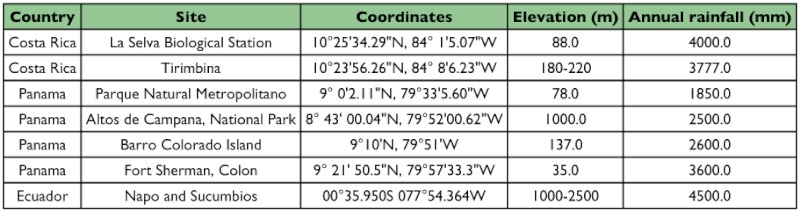
Descriptions of collecting sites.

Caterpillars in the genus *Eois* are the most frequently encountered herbivores on *Piper*. Eois is one of the largest genera in the subfamily Larentiinae (Geometridae) and is noted for its species richness with 257 species reported worldwide (Scoble 1999; Brehm and Fiedler 2005) although many more likely await discovery. The associated distribution of *Eois* and *Piper* extends from Mexico to Chile and Argentina (Scoble 1999), where they occur in lowland and highland forests across a range of climatic conditions. *Eois* are generally considered to be weak dispersers (Brehm 2000) and caterpillars often occur at densities causing high levels of damage; sometimes completely defoliating young plants (all authors, pers. obs.). *Eois* caterpillars are relatively small, approximately 13–20 mm in their final instar and are mostly cryptic in coloration. Parasitoids are important agents of mortality for insect larvae (Hawkins et al. 1997) and *Eois* are attacked by a suite of parasitoids including wasps primarily in the families, Braconidae and Ichneumonidae and flies in the family Tachinidae.

### Study sites

Caterpillars were collected and reared from forests in Costa Rica, Panama and Ecuador that represent geographic regions that are separated by large distances and have a distinct evolutionary and geological history, including the rising of the Isthmus of Panama and the formation of the Ecuadorian Andes. These forests differed in elevation and climate, and were surveyed for different durations (see Table 1 for site details).

### Collection methods

Caterpillar collections were carried out in Costa Rica from 1995–2007, Ecuador from 2000–2007 and in Panama from 2003 to 2005. In Costa Rica and Ecuador, caterpillars were collected from *Piper* plants either haphazardly, or by intensive sampling within 10m diameter plots. Leaves from individual *Piper* plants were collected and surveyed for caterpillars or eggs of *Eois*. In Panama, sampling was conducted by examining *Piper* plants present at each site, using fresh leaf damage and frass as cues for locating *Eois* caterpillars. On Barro Colorado Island, extensive searches were conducted on *Piper aequale* Vahl. and *Piper schiedeanum* Steud., since *Eois* are abundant on these plants. Only third to fifth (final) instars were included in statistical analyses across study sites.

Caterpillars were reared in plastic bags or cups in ambient conditions and fed fresh leaves as needed until pupation. Each caterpillar collected was assigned a unique number and logged into a database; recorded information included hostplant species, larval stage, collection date, locale, and for Ecuador, the site and altitude of the plot. *Piper* plants and *Eois* caterpillars were identified either to species or to morphospecies. We created digital images of *Eois* caterpillars throughout their development and recorded changes in coloration between instars to assist in morphospecies identification. Voucher specimens of *Eois* adults are deposited at the Smithsonian Institute, while parasitoids are in the collections of J. Stireman (Tachinidae; Wright State University), J. Whitfield (Braconidae: Microgasterinae; University of Illinois), and S. Shaw (Meteorinae and Ichneumonidae; University of Wyoming).

### Statistical Analyses

SAS (9.1) statistical software was used for all analyses. Associations between incidence of parasitism and categorical abiotic variables were assessed using logit models and two-way contingency tables. Incidence of parasitism (a dichotomous variable) included only data for which either a parasitoid or a moth emerged from a collected caterpillar. Levels of parasitism were calculated as the percentage of parasitoids reared from the total number of results (emerged adults and parasitoids). For analyses of parasitoid diversity, parasitoid wasps were separated by family as they exhibit a range of differences in their feeding ecology (Harvey and Strand 2002).

Hostplant specificity of *Eois* caterpillars was quantified using several methods. The traditional measure is the average number of plant species on which each *Eois* morphospecies was collected. This measure was supplemented with a Chao2 estimate, (Colwell and Coddington 1994) to estimate the number of *Piper* host species expected for an *Eois* species. Chao2 provides good predictive power for presence-absence data with small sample sizes. We also use modifications of Whittaker's index of χdiversity as a proxy for measuring specialization (Dyer et al. 2007) across *Piper* species. Specialization at the level of plant family was compared between *Eois* and other Neotropical Lepidoptera larvae using modifications of Whittaker's χdiversity index.

## Results

**Figure 1.**
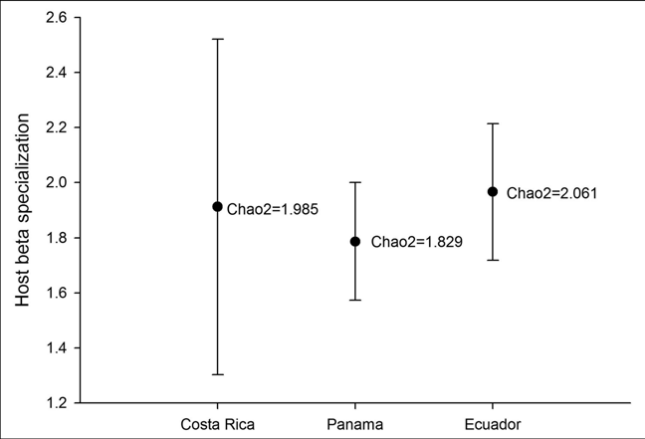
Host beta specialization across geographic regions calculated as the mean number of *Piper* species per *Eois* species. Chao2 is an additional measure of host specificity used to estimate the number of *Piper* host species expected for each species of *Eois*. Error bars represent 95% confidence intervals.

**Figure 2.**
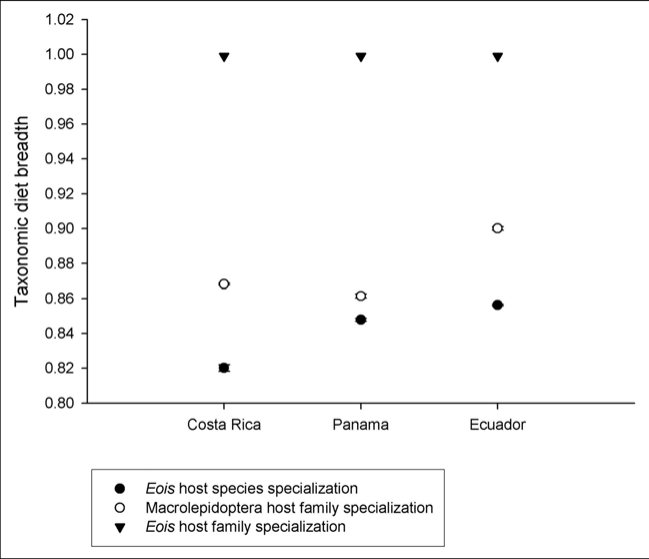
Diet breadth calculated as *Eois* species turnover with *Piper* species using Whitakers χas a measure of hostplant specialization (closed circles). Higher values of χindicate a greater degree of specialization. Error bars represent 95% confidence intervals. Open circles and triangles represent diet breadth of macrolepidoptera and *Eois* at the level of plant family. A value of 1 represents the maximum level of host specificity.

Our Neotropical rearing database, which includes over 50,000 individual plant-caterpillar collections representing 95 plant families and 29 families of Lepidoptera, has produced 15,034 records for *Eois* (see Table 2 for *Piper* species). The results reveal that *Eois* is associated exclusively with *Piper*, and furthermore, that each *Eois* species is restricted to an average of approximately 2 species of *Piper* (Figure 1). We have reared 72 *Eois* morphospecies from Ecuador, 23 from Costa Rica and 6 from Panama. These were collected on 72 species of *Piper* (42 known species and 30 morphospecies). When we compared *Eois* species turnover per *Piper* species across geographic regions we found that values of host specificity are marginally greater in Ecuador than in Panama and Costa Rica (Figure 2). We also contrasted *Eois* hostplant specificity at the family level with data collected for other Macrolepidoptera. We obtained xdiversity values of 1 for *Eois* and 0.86–0.95 for the remaining Macrolepidoptera, where 1 represents the highest level of host specificity (association with a single family).

**Table 2.**
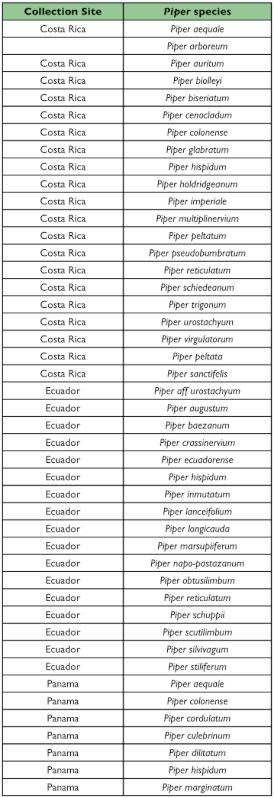
List of *Piper* plants sampled that are known to species

**con't:**
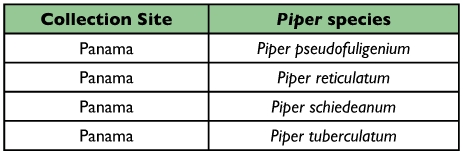

In the lowlands (below 1,000m), *Eois* development from 1^st^ instar until pupation takes 9–10 days, with an additional 12 days until adult eclosure. In the highlands (above 1,000m), *Eois* development from 1^st^ instar until pupation takes approximately 29–30 days, with 23 days until adult eclosure. A total of 1,038 parasitoid individuals were reared from *Eois*, representing three wasp families, Braconidae (N=883), Eulophidae (N= 12) and Ichneumonidae (N=81) as well as parasitoid flies in the family Tachinidae (N= 76). Because we did not include 1^st^ and 2^nd^ instars in our analyses, these results underestimate parasitism. Parasitoid wasps usually emerged from 3^rd^ instar caterpillars, whereas tachinids most frequently emerged from pupae. Parasitoids were primarily solitary, with a single adult emerging from each caterpillar.

**Figure 3.**
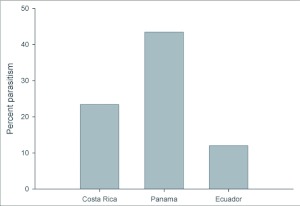
Geographic variation in percent parasitism of *Eois*. Ecuador (N=3440, 8 years; 2000–2007), Panama (N= 1777, 3 years; 2003–2005), Costa Rica (N= 1874, 12 years; 1995–2007). Incidence of parasitism varied significantly among geographic regions (DF = 2, χ^2^= 472.05, P < 0.0001) with greater parasitism of *Eois* in Panama.

**Figure 4.**
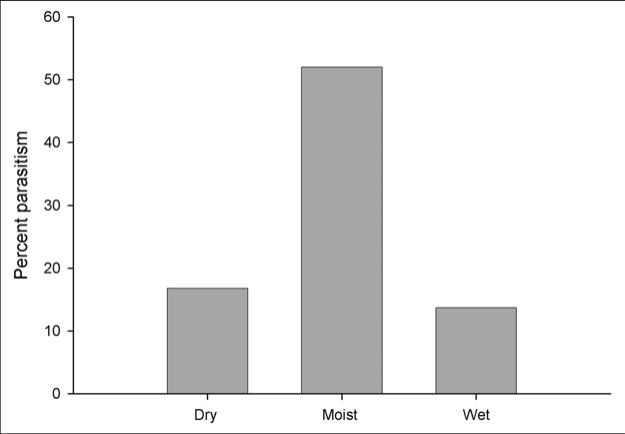
Percent parasitism of *Eois* across a precipitation gradient. Dry forest (N=351 ), moist forest (N= 1227) and wet forest (N=4460). Incidence of parasitism of *Eois* varied significantly with precipitation (DF = 2, χ^2^ = 830.3, P < 0.0001) with greater parasitism in the moist forest.

Across geographic regions, parasitism rates of *Eois* varied significantly, with the highest rates observed in Panama (43.48%), Mowed by Costa Rica (23.41%) and Ecuador (12.02%) (Figure 3) (DF = 2, χ^2^ = 472.05, P < 0.0001). Parasitism was significantly correlated with precipitation; higher rates were found in moist (52%) versus dry (16.8%) and wet forests (13.7%) (Figure 4) (DF = 2, χ^2^ = 830.3, P < 0.0001). Parasitism was also significantly higher at low (< 1000m; 35.23%) compared to higher elevations (10.5%; >1000–2500m+; DF = 1, χ^2^ = 539.6, P < 0.0001). The logit model revealed that precipitation was the best predictor of parasitism rate (Table 3).

**Figure 5.**
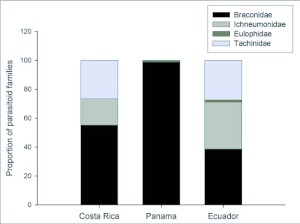
Geographic variation in the proportion of parasitoid families reared from *Eois*. Costa Rica (Braconidae N=59, lchneumonidae N=7, Tachinidae N=10), Ecuador (Braconidae N= 141, lchneumonidae N=74, Eulophidae N=4, Tachinidae N=64) and Panama (Braconidae N=683, Eulophidae N=8, Tachinidae N=2). Parasitoid family diversity varied significantly among geographic regions (DF = 4, χ^2^=401.9, P < 0.0001) and was greater in Ecuador compared to Costa Rica and Panama where parasitoids were primarily from the family Braconidae.

Our analysis shows that *Eois* are generally attacked by the same parasitoid families across geographic regions, but there were significant differences in the proportion of parasitoid families reared from *Eois* (Figure 5) (DF = 4, χ^2^ =401.9, P < 0.0001), with greater diversity occurring in Ecuador compared to Panama and Costa Rica, where parasitoids were primarily from the family Braconidae (Figure 5). Parasitoid family diversity was also significantly greater in wet versus dry forests (DF= 4, χ^2^= 320.1, P<0.0001) and at high versus low elevations (DF =1 χ^2^= 400.5, P < 0.0001). Furthermore, 64 of 76 tachinids and 74 of 81 ichneumonids were reared in the highlands, whereas 742 of 883 braconids were reared from the lowlands. Tachinids and ichneumonids were reared almost exclusively from wet forests, with the exception of 2 tachinids that were reared from a moist forest site (Panama). Braconids were reared from dry (N=58), moist (N=625) and wet forests (N=200).

## Discussion

Our research is unique in that it is based on a large database documenting interactions within a single caterpillar genus, feeding on the same hostplant genus across a broad geographic range. We have demonstrated that *Eois* larvae exhibit high levels of hostplant specialization (Figure 2). In terms of the relative degree of host specificity within *Piper*, our results did not support the hypothesis that host specificity varies geographically, as each species of *Eois* has a narrow diet breadth, usually including just one or two *Piper* species (Figure 1).

**Table 3.**
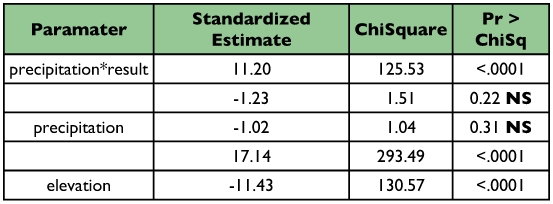
Analysis of Maximum Likelihood Estimates

Despite the lack of variation in *Eois* diet breadth across our study sites, a mosaic of potential selective pressures from parasitism exists. First, we found support for our hypothesis that parasitism rates are higher at low elevations. Interestingly, parasitism rates of *Eois* were 3 times higher at low compared to high elevations, despite the fact that the caterpillars developed more rapidly in the lowlands (9–10 days to pupal stage versus 29–30 days at higher elevations). Our results do not provide support for the slow growth high mortality hypothesis (Clancy and Price 1987), since slow development was not associated with increased vulnerability to parasitoids. Second, *Eois* are exposed to striking differences in parasitism rates with seasonal variation in rainfall and their susceptibility appears to be greatest in moist tropical forests (Figure 4). Thus, we did not find support for the hypothesis that parasitism is higher in wet forests. Whether these differences are related to interactions with parasitoids and plant chemistry, variation in plant quality on caterpillar development or climatic effects on parasitoids in dry and wet forests remains to be explored. There is evidence that young leaves are eaten more rapidly in the moist forest on BCI than in the drier Brazilian Cerrado or Mexican dry forest, and both young and mature leaves are eaten more rapidly on BCI than at the high rainfall Costa Rican forest, La Selva (Leigh et al. 2004). It appears that, along a gradient of increasing precipitation, there is a peak in the consumption rate of young leaves in transitional forests with dry seasons of intermediate length (Marquis et al. 2002). Therefore, seasonality may provide an important peak in caterpillar abundance for parasitoid foraging in moist versus dry forests where parasitoids may be more vulnerable to desiccation (Shapiro and Pickering 2000). It is however currently unknown whether *Eois* are more abundant in moist versus dry and wet forests.

The comparison across geographic regions revealed that parasitism rates are significantly greater in Panama compared to Costa Rica and Ecuador (Figure 3). This pattern may be due to the lower diversity of parasitoid families observed in Panama (Figure 5). Similar to our study, Rodriguez and Hawkins (2000) also found higher rates of parasitism associated with lower parasitoid diversity. In Panama, caterpillars were primarily parasitized by braconids compared to Costa Rica and Ecuador where Tachinidae and Ichneumonidae were also reared from *Eois*. Why tachinids and ichneumonids were virtually absent from Panama is unclear. At least for tachinids, this pattern is not due to low abundance; equal proportions of parasitoid wasps and flies were reared from macrolepidopteran larvae in the same Panamanian forests from which *Eois* were also collected (dry forest, 53% versus 47%; wet forest, 51% versus 49% for wasps and flies respectively) (Connahs unpublished). There are likely to be a multitude of factors that vary across these sites, minimizing inferences that can be made about parasitoid foraging and behavior without further investigation.

Although we found significant variation in parasitism of *Eois*, measures of host specificity did not vary greatly across this broad geographic scale. While these results may suggest that parasitoids do not influence *Eois* specialization, other records indicate that incidence of parasitism can vary significantly depending on the particular *Piper* host species (authors, unpublished data). Thus, it may be difficult to examine factors driving herbivore specialization on such a large spatial scale.

The radiation of *Piper* in the Neotropics appears to have been accompanied by high levels of *Eois* species diversity, particularly in the Andes (personal observations, all authors). *Piper* represents a dominant Neotropical plant genus (Gentry 1993); therefore this association may represent one of the most pervasive plant-herbivore interactions in tropical forests. We propose that the *Piper-Eois* system is an ideal model for addressing coevolutionary hypotheses for several reasons. First, we have demonstrated that *Eois* species are extremely host specific across a broad geographic scale. Second, *Piper* is known to contain metabolites toxic to generalist herbivores (Batista-Perieira et al. 2006, Fincher et al. 2008, Vanin et al. 2008), yet *Eois* appear to have overcome many of these toxins, and can completely defoliate particular species of *Piper*. Finally, many species of *Eois* are well camouflaged on *Piper* and exhibit specialized defensive behaviors such as régurgitation if disturbed. Regurgitation is an important defense for lepidopteran larvae (Gentry and Dyer 2002) and may contain toxic compounds repellent to parasitoids (Feltwell 1982). Preliminary data suggests that some *Eois* species sequester *Piper* compounds (Dyer et al. 2004a, Connahs unpublished), which could be used for defense. We suggest that reciprocal coevolution may be occurring between *Piper* and *Eois*, and is potentially mediated by variation in parasitoid pressure.

In order to adequately address questions regarding the relative importance of top down forces on the evolution of *Eois* diet breadth, more data are required on the phylogenetic relationships between *Piper, Eois* and their parasitoids. As Bernays and Graham (1988) pointed out, plant-insect interactions are ecologically dynamic and current associations do not represent the end-point of coevolutionary processes that have occurred over millions of years. *Piper* is an ancient genus Jaramillo 2006,) thus, uncovering the age of *Eois*' association with *Piper* would open up exciting opportunities for testing hypotheses on evolutionary mechanisms driving plant and insect diversity in tropical forests. The tropics are home to an extraordinary array of specialized relationships between plants and animals and as the natural history of these systems becomes better resolved, more interesting specialization stories are likely to emerge.

## Editor's note

Paper copies of this article will be deposited in the following libraries. Senckenberg Library, Frankfurt Germany; National Museum of Natural History, Paris, France; Field Museum of Natural History, Chicago, Illinois USA; the University of Wisconsin, Madison, USA; the University of Arizona, Tucson, Arizona USA; Smithsonian Institution Libraries, Washington D.C. USA; The Linnean Society, London, England.
